# Do the Rich Always Become Richer? Characterizing the Leaf Physiological Response of the High-Yielding Rice Cultivar Takanari to Free-Air CO_2_ Enrichment

**DOI:** 10.1093/pcp/pcu009

**Published:** 2014-01-30

**Authors:** Charles P. Chen, Hidemitsu Sakai, Takeshi Tokida, Yasuhiro Usui, Hirofumi Nakamura, Toshihiro Hasegawa

**Affiliations:** ^1^Agro-Meteorology Division, National Institute for Agro-Environmental Sciences, 3-1-3 Kannondai, Tsukuba, Ibaraki, 305-8604 Japan; ^2^Carbon and Nutrient Cycling Division, National Institute for Agro-Environmental Sciences, 3-1-3 Kannondai, Tsukuba, Ibaraki, 305-8604 Japan; ^3^Taiyo-Keiki Co., Ltd., 1-12-3 Nakajujo, Kita, Tokyo, 114-0032 Japan; ^4^Present address: Department of Biology and Chemistry, Azusa Pacific University, 901 E. Alosta Avenue, Azusa, CA 91702, USA.

**Keywords:** Ecophysiology, FACE, Global change, Photosynthesis

## Abstract

The development of crops which are well suited to growth under future environmental conditions such as higher atmospheric CO_2_ concentrations ([CO_2_]) is essential to meeting the challenge of ensuring food security in the face of the growing human population and changing climate. A high-yielding *indica* rice variety (*Oryza sativa* L. cv. Takanari) has been recently identified as a potential candidate for such breeding, due to its high productivity in present [CO_2_]. To test if it could further increase its productivity under elevated [CO_2_] (eCO_2_), Takanari was grown in the paddy field under season-long free-air CO_2_ enrichment (FACE, approximately 200 µmol mol^−1^ above ambient [CO_2_]) and its leaf physiology was compared with the representative *japonica* variety ‘Koshihikari’. Takanari showed consistently higher midday photosynthesis and stomatal conductance than Koshihikari under both ambient and FACE growth conditions over 2 years. Maximum ribulose-1,5-bisphosphate carboxylation and electron transport rates were higher for Takanari at the mid-grain filling stage in both years. Mesophyll conductance was higher in Takanari than in Koshihikari at the late grain-filling stage. In contrast to Koshihikari, Takanari grown under FACE conditions showed no decrease in total leaf nitrogen on an area basis relative to ambient-grown plants. Chl content was higher in Takanari than in Koshihikari at the same leaf nitrogen level. These results indicate that Takanari maintains its superiority over Koshihikari in regards to its leaf-level productivity when grown in elevated [CO_2_] and it may be a valuable resource for rice breeding programs which seek to increase crop productivity under current and future [CO_2_].

## Introduction

As the world population continues to increase in the coming decades, the demand for food is expected to increase substantially (Alexandratos and Bruinsma 2012). However, the progression of climate change and an increasing frequency of extreme weather events in various parts of the world are expected to have negative effects on food crop yields in the future (Hatfield et al. 2011, Yadav et al. 2011). To ensure food security going forward, it is essential that we gain an understanding of how crops respond to the changing environment and use that information to develop new varieties of staple food crops which can tolerate future conditions or even take advantage of them to become more productive than current cultivars (Ainsworth et al. 2008, Ziska et al. 2012, Tausz et al. 2013).

The rise of atmospheric concentrations of CO_2_ ([CO_2_]) since the Industrial Revolution is one of the most clearly documented aspects of global change, and the global mean [CO_2_] is projected to continue to rise over the course of this century (Meehl et al. 2007). The primary effect of elevated [CO_2_] (eCO_2_) on the physiology of C_3_ crops is an increase in the rate of photosynthesis, although this does not always translate into an equivalent increase in biomass accumulation or yield (Leakey et al. 2009).

Rice (*Oryza sativa* L.) is the staple food for over half of the world’s population, and by 2050 the demand for rice is expected to increase by nearly 30% over 2005–2007 production levels (Alexandratos and Bruinsma 2012). The genetic diversity of rice is high, and improving its yield potential via selective breeding of key cultivars will be a large step towards achieving higher productivity in the future (Khush 2005).

A high-yielding *indica* variety of rice called ‘Takanari’ was developed in Japan in the 1980s as the offspring of two high-yielding cultivars from Korea (‘Milyang 25’ and ‘Milyang 42’) (Imbe et al. 2004, Takai et al. 2012). Compared with the average *japonica* rice variety, Takanari exhibits some remarkable physiological characteristics, such as a very high stomatal and hydraulic conductance, as well as a large below-ground rooting system which enables it to accumulate a large amount of nitrogen (N) (Taylaran et al. 2009, Taylaran et al. 2011). These physiological traits result in high source capacity (i.e. leaf-level photosynthesis and carbon assimilation) (Taylaran et al. 2011) and, coupled with a high sink capacity (i.e. large panicle size and excellent grain-filling efficiency) (Nagata et al. 2001, Hirasawa et al. 2010), are responsible for its high biomass and yield when grown under present-day field conditions. Unfortunately, the quality and edibility of its grain are low (Imbe et al. 2004), but it remains a potential germplasm resource for breeding higher productivity into the next generation of modern rice varieties.

Consequently, to examine its response under future atmospheric conditions in the field, Takanari has been grown at two free-air CO_2_ enrichment (FACE) experimental sites in Japan under a season-long open-air fumigation of elevated [CO_2_] (+200 µmol mol^−1^ above ambient [CO_2_]) (Hasegawa et al. 2013). FACE technology involves the computer-controlled release of CO_2_ from an array of pipes or blowers laid out in the field; the gas is then carried across the treatment area via natural wind and diffusion (Hendrey and Miglietta 2006). It has been used for >20 years to investigate the response of a variety of natural and managed ecosystems to eCO_2_ (reviewed by Ainsworth and Long 2005, Nösberger et al. 2006).

At the Shizukuishi FACE site in northeastern Japan during the 2008 growing season, Takanari showed a 16% yield enhancement in eCO_2_, compared with 10% for ‘Koshihikari’ (currently considered the representative *japonica* variety, and grown widely across Japan) (Hasegawa et al. 2013). In 2010, at the Tsukubamirai FACE site in central Japan, Takanari showed a 21% yield enhancement in eCO_2_ compared with 16% for Koshihikari (Hasegawa et al. 2013). The brown rice yield (g m^−2^) of Takanari was 26% and 32% higher than that of Koshihikari under ambient and eCO_2_ conditions, respectively, indicating that the large sink capacity of Takanari (specifically, high grain number per panicle and high panicle number per hill) was effective in enabling a strong yield enhancement response in eCO_2_ (Hasegawa et al. 2013). However, the response of the source capacity (i.e. photosynthesis) and the leaf-level physiology of Takanari under eCO_2_ have not yet been examined.

The objective of this study was to characterize and compare the leaf-level physiology of the rice cultivars Takanari and Koshihikari under a season-long, free-air elevated [CO_2_] treatment. We sought to answer the question of whether the source capacity of Takanari during grain filling is greater than that of Koshihikari in eCO_2_, by examining the photosynthesis, the stomatal and mesophyll conductance, leaf N, protein content and pigment content of the two cultivars from the booting stage to the late grain-filling (GF) stage over parts of two growing seasons.

## Results

### Photosynthesis and stomatal conductance

**Fig. 1:**
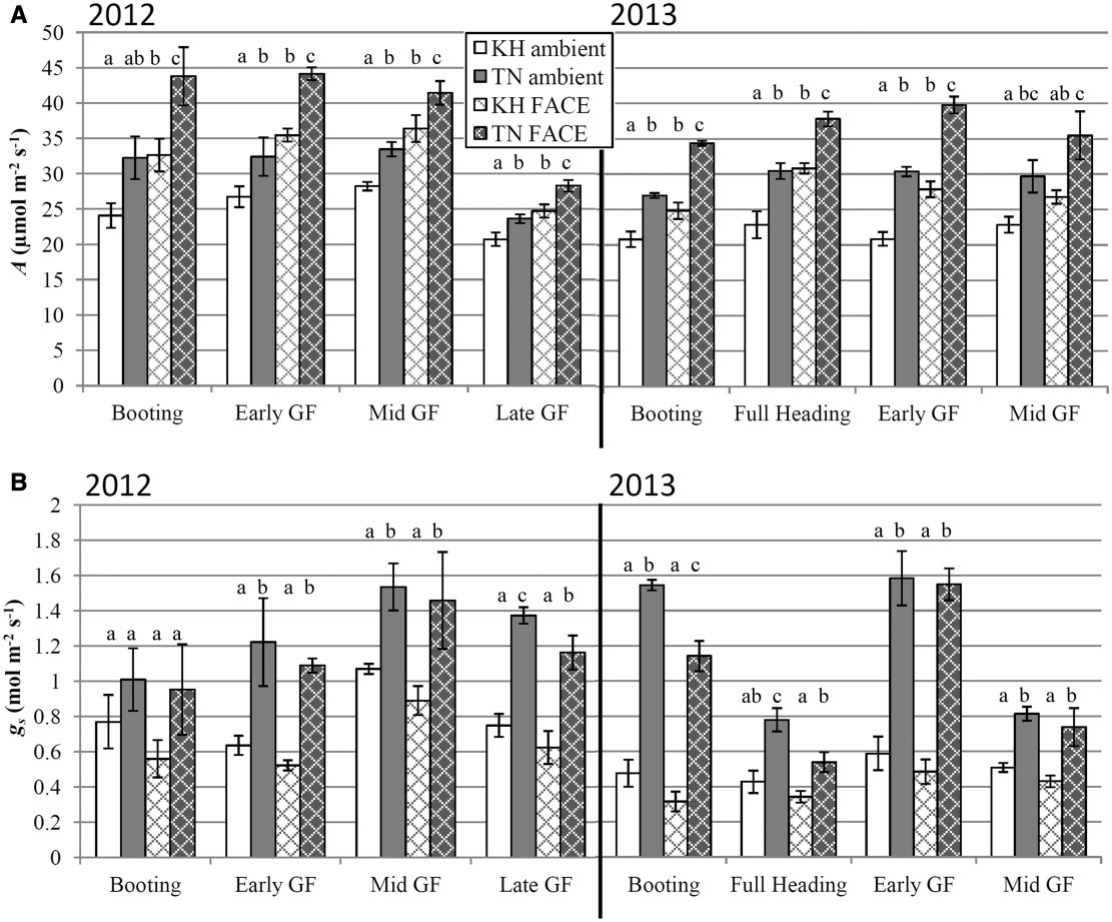
Midday photosynthetic gas exchange measurements taken at the respective growth [CO_2_] for ambient (plain bars) and FACE (cross-hatched bars) treatments (390 and 590 µmol mol^−1^, respectively). Koshihikari (KH) is shown in the white bars and Takanari (TN) in the gray bars. Data are from the 2012 and 2013 field seasons. The measurements were taken at the following growth stages: (1) booting (approximately 1.5 weeks before heading); (2) full heading (3 d after heading, 2013 only); (3) early grain filling (GF) stage (1 week after heading); (4) mid GF stage (2 weeks after heading); and (5) late GF stage (4 weeks after heading, 2012 only). All measurements were taken on flag leaves. Error bars are SEMs. Different letters above each bar indicate significant differences between means at α = 0.05 within each measurement time point. *n* = 4. (A) Net photosynthetic carbon assimilation rate (*A*). (B) Stomatal conductance to water vapor (*g*_s_).

The photosynthetic rate (*A*) of Takanari flag leaves was significantly higher than that of Koshihikari in both the ambient and eCO_2_ treatments across all measurement time points in 2012 and 2013, except for the ambient-grown plants at the booting stage (1.5 weeks before heading) in 2012 (Fig. 1A; Table 1). The highest photosynthetic rate for Takanari flag leaves in the FACE treatment (at 590 µmol mol^−1^) was 44.2 µmol CO_2_ m^−2^ s^−1^, observed at the early GF stage (1 week after heading) in 2012. In comparison, the maximum observed midday photosynthesis of Koshihikari in FACE was 36.4 µmol CO_2_ m^−2^ s^−1^ at the mid GF stage (2 weeks after heading) in 2012. The relative advantage of Takanari over Koshihikari at each growth stage was generally similar between the ambient and FACE treatments. For example, during the booting stage in 2012, *A* of Takanari was 34.0% and 34.2% higher than that of Koshihikari in the ambient and FACE treatments, respectively. This advantage in Takanari decreased over time to 14.0% and 14.4% at the late GF stage (4 weeks after heading) in 2012, resulting in a significant cultivar by stage effect for *A* in 2012 (*P* = 0.046, Table 1). In 2013, *A* in Takanari was consistently higher than in Koshihikari from booting to mid GF (2 weeks after heading) in both ambient and FACE treatments, but the highest advantage of Takanari was observed at early GF (1 week after heading) (45.7% and 42.7%, respectively).

**Table 1:** Three-way analysis of variance of the midday net photosynthetic carbon assimilation rate (*A*), stomatal conductance to water vapor (*g*_s_), maximum carboxylation rate of Rubisco (*V*_c,max_), maximum electron transport rate (*J*_max_), mesophyll conductance to CO_2_ (*g*_m_), stomatal limitation to photosynthesis (SL) at each treatment’s respective growth [CO_2_] and mesophyll conductance limitation to photosynthesis (MCL) at each treatment’s respective growth [CO_2_] from Figs. 1 and 2

Effect	*A*	*g*_s_	*V*_c,max_	*J*_max_	*g*_m_	SL	MCL
**2012 growing season**							
Cultivar	**<0.0001**	**<0.0001**	**0.0033**	**<0.0001**	**0.0002**	**0.0003**	**0.0005**
Growth CO_2_	**<0.0001**	**0.0191**	NS	**0.0134**	**0.0018**	**<0.0001**	**<0.0001**
Stage	**<0.0001**	**<0.0001**	**<0.0001**	**0.0321**	**<0.0001**	NS	**<0.0001**
Cultivar × CO_2_	NS	NS	NS	NS	NS	NS	NS
Cultivar × Stage	**0.0457**	NS	**0.0207**	NS	NS	NS	**0.0084**
CO_2_ × Stage	**0.0498**	NS	NS	NS	NS	NS	NS
Cultivar × CO_2_ × Stage	NS	NS	NS	NS	NS	NS	NS
2013 growing season							
Cultivar	**<0.0001**	**<0.0001**	**<0.0001**	**0.0117**	**0.0005**	**0.0005**	*0.062*
Growth CO_2_	**<0.0001**	**0.0003**	NS	**0.0021**	**0.0023**	**0.0002**	**<0.0001**
Stage	**0.0039**	**<0.0001**	**0.0149**	**<0.0001**	**0.0003**	**<0.0001**	NS
Cultivar × CO_2_	NS	NS	NS	NS	NS	NS	NS
Cultivar × Stage	NS	**<0.0001**	NS	*0.087*	**0.0025**	NS	NS
CO_2_ × Stage	NS	NS	NS	NS	NS	NS	NS
Cultivar × CO_2_ × Stage	NS	NS	NS	NS	NS	NS	*0.082*

Data are from the 2012 and 2013 growing seasons.

*P*-values <0.05 are in bold; *P*-values >0.05 but <0.10 are in italics; *P*-values >0.10 are indicated by NS.

The overall effect of FACE on photosynthesis was significant in both years (Fig. 1A; Table 1). The relative enhancement of *A* in plants grown in FACE over ambient conditions was comparable in the two cultivars within each growth stage. In 2012, the enhancement of *A* by FACE at the booting stage in Koshihikari and Takanari was 35.6% and 35.8%, respectively; this decreased to 19.3% and 19.7% at the late GF stage. During the 2013 field season, the enhancement of *A* by FACE ranged from 17.1% to 35.0% in Koshihikari and from 19.5% to 30.9% in Takanari.

Takanari consistently showed a significantly higher stomatal conductance (*g*_s_) than that of Koshihikari, regardless of the growth stage or growth [CO_2_] in both years, with the exception of the booting stage in 2012 (Fig. 1B; Table 1). The difference in *g*_s_ between cultivars varied between measurement time points, but *g*_s_ reached as high as approximately 100% and 195% higher in Takanari than in Koshihikari at early GF in 2012 and 2013, respectively. There was a significant overall effect of FACE on *g*_s_ over the entire experiment in both years (*P* = 0.019, 0.0003; Table 1), but this FACE-induced decrease in *g*_s_ was only significant at the level of individual treatment mean comparisons in Takanari at the late GF in 2012 and at booting and full heading (3 d after heading) in 2013 (Fig. 1B).

### Biochemical capacity for photosynthesis

**Fig. 2:**
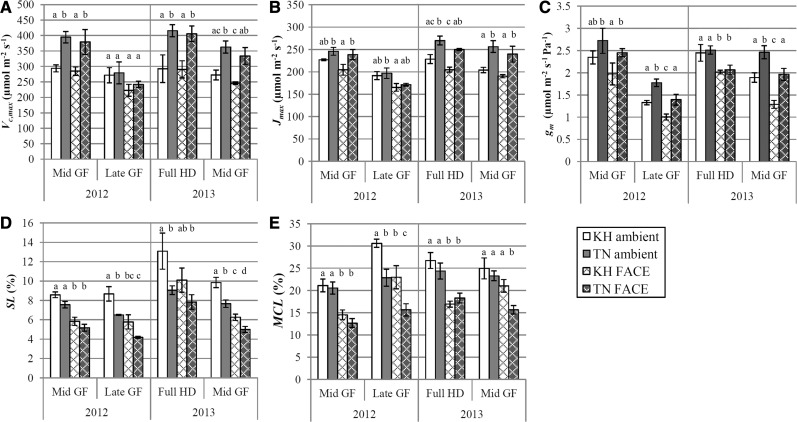
Photosynthetic parameters derived from analysis of CO_2_ response curves of Koshihikari (white bars) and Takanari (gray bars) grown under ambient (plain bars) and FACE (cross-hatched bars) conditions. Data were taken in 2012 at the mid grain filling (GF) (2 weeks after heading) and late GF stage (4 weeks after heading), and in 2013 at full heading (HD) (3 d after heading) and the mid GF stage. All measurements were taken on flag leaves. Error bars are SEMs. Different letters above each bar indicate significant differences between means at α = 0.05 within each measurement time point. *n* = 4. (A) Maximum carboxylation rate of Rubisco (*V*_c,max_). (B) Maximum electron transport rate (*J*_max_). (C) Mesophyll conductance to CO_2_ (*g*_m_), expressed in µmol m^−2^ s^−1^ Pa^−1^; when atmospheric pressure is 101.3 kPa, this is nearly equal to 10 times the value of *g*_m_ expressed in mol m^−2^ s^−1^. (D) Stomatal limitation to photosynthesis (SL) at each treatment’s respective growth [CO_2_]. (E) Mesophyll conductance limitation to photosynthesis (MCL) at each treatment’s respective growth [CO_2_].

Takanari showed a significantly higher biochemical capacity for photosynthesis than Koshihikari, as indicated by higher values of *V*_c,max_ [the maximum carboxylation rate of ribulose-1,5-bisphosphate carboxylase oxygenase (Rubisco)] and *J*_max_ (the maximum electron transport rate supporting the regeneration of ribulose-1,5-bisphosphate) (Fig. 2A, B; Table 1). *V*_c,max_ and *J*_max_ were both higher in Takanari than in Koshihikari at the full heading stage in 2013 and mid GF in 2012 and 2013. However, at late GF in 2012, there was no significant difference in *V*_c,max_ and *J*_max_ between either cultivars or CO_2_ treatment.

### CO_2_ diffusion-related limitations to photosynthesis

There was a significant overall effect of cultivar, growth [CO_2_] and stage on mesophyll conductance (*g*_m_) in both 2012 and 2013 (Table 1). During the 2012 growing season, Takanari had a higher *g*_m_ than Koshihikari in the FACE treatment at mid GF and in both ambient and FACE treatments at late GF (Fig. 2C). In 2013, *g*_m_ was identical between cultivars at full heading but was higher in Takanari than in Koshihikari at mid GF due to a decrease in *g*_m_ in the latter. Flag leaves in the FACE treatment generally showed lower *g*_m_ than in the ambient treatment in both years.

Stomatal limitation to photosynthesis (SL) and mesophyll conductance limitation to photosynthesis (MCL) were calculated for each CO_2_ response curve at the growth [CO_2_] (*C*_a_ = 390 and 590 µmol mol^−1^ for ambient and FACE-grown plants, respectively) using Equations 1 and 2 (see the Materials and Methods and Supplementary Fig. S2 for details). Values of SL ranged from 4.2% to 8.6% over the mid and late GF stages in 2012, and from 5.0% to 13.1% over the full heading and mid GF stages in 2013 (Fig. 2D). There was a significant overall cultivar effect for SL in both years (Table 1). SL was significantly lower in Takanari than in Koshihikari in the ambient treatment at late GF in 2012 and full heading and mid GF in 2013 (Fig. 2D). SL in the FACE treatment was significantly lower than ambient treatment in both years (Fig. 2D).

Values of MCL ranged from 12.7% to 30.6% in 2012 and from 15.6% to 26.7% in 2013. Overall, MCL was lower in FACE than in the ambient treatments (Fig. 2E). There was no difference in MCL between cultivars at mid GF in 2012 and full heading in 2013, but MCL was significantly lower in Takanari than in Koshihikari at late GF in 2012 for both CO_2_ treatments and at mid GF in 2013 for the FACE treatment (Fig. 2E).

### Leaf protein, nitrogen and photosynthetic pigment content

**Fig. 3:**
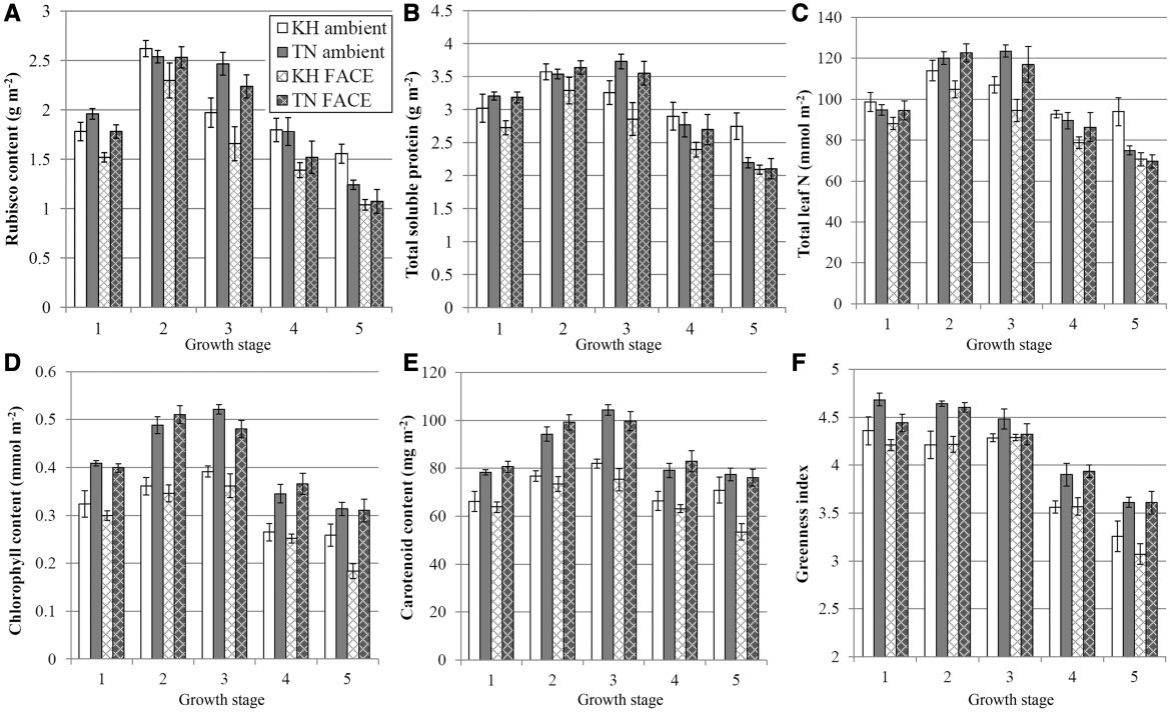
Leaf protein, nitrogen and pigment content of Koshihikari (white bars) and Takanari (gray bars) grown under ambient (plain bars) and FACE (cross-hatched bars) conditions. Flag leaves were sampled in 2012 at the following five growth stages: (1) booting (1.5 weeks before heading); (2) early grain filling (GF) stage (1 week after heading); (3) mid GF stage (2 weeks after heading); (4) mid-late GF stage (3 weeks after heading); and (5) late GF stage (4 weeks after heading). Error bars are SEMs. *N* = 4. (A) Rubisco content. (B) Total leaf soluble protein. (C) Total leaf nitrogen. (D) Total Chl (*a* + *b*). (E) Carotenoid content. (F) Greenness index (total Chl/carotenoid).

The uppermost fully expanded leaves were sampled at several time points from the booting stage (late July) to late GF stage (early September) in the 2012 growing season. For total Rubisco, leaf soluble protein and N content, there was a clear effect of growth stage on the values in both cultivars (Fig. 3A–C; Table 2). Values increased and peaked shortly after heading, and steadily decreased afterwards. In both cultivars, the peak values of Rubisco, soluble protein and leaf N were observed at early GF (1 week after heading), but Takanari had significantly higher values than Koshihikari at mid GF (2 weeks after heading) (Fig. 3A–C).

**Table 2:** Three-way analysis of variance of the time course data for Rubisco content, leaf soluble protein, total leaf nitrogen content, total Chl (*a* + *b*) content, carotenoid content and greenness index from Fig. 3

Effect	Rubisco content	Soluble protein	Total leaf N	Total Chl	Carotenoid	Greenness index
Cultivar	**0.0018**	**0.0079**	**0.007**	**<0.0001**	**<0.0001**	**<0.0001**
Growth CO_2_	**0.0127**	NS	NS	NS	NS	NS
Stage	**<0.0001**	**<0.0001**	**<0.0001**	**<0.0001**	**<0.0001**	**<0.0001**
Cultivar × CO_2_	**0.0361**	**0.0048**	**0.0026**	**0.0289**	**0.0022**	NS
Cultivar × Stage	**0.0004**	**0.002**	**<0.0001**	**0.0367**	*0.0571*	NS
CO_2_ × Stage	NS	NS	NS	NS	**0.0306**	NS
Cultivar × CO_2_ × Stage	NS	NS	NS	NS	NS	NS

Data are from the 2012 growing season.

*P*-values <0.05 are in bold; *P*-values >0.05 but <0.10 are in italics; *P*-values >0.10 are indicated by NS.

There was a significant cultivar by growth CO_2_ interaction effect for Rubisco, soluble protein and total leaf N content across all the time points as a whole (*P* = 0.036, 0.005 and 0.003, respectively; Table 2). In Koshihikari, Rubisco, soluble protein and leaf N content were significantly decreased under the FACE treatment over the entire sampling period. In contrast, in Takanari, there was no difference in Rubisco, total soluble protein and leaf N content between ambient- and FACE-grown leaves.

Total Chl (Chl *a* + *b*) and carotenoid content was significantly higher in Takanari than in Koshihikari across the entire sampling period (Fig. 3D, E; Table 2). On average, Takanari had 29.3% and 45.5% higher Chl content than Koshihikari in the ambient and FACE treatments, respectively. In both cultivars, the Chl and carotenoid content reached a peak at mid GF and decreased thereafter.

The greenness index, an indicator of leaf senescence, was about 4.3–4.6 for both cultivars from booting stage until mid GF, after which it fell sharply (Fig. 3F). From the mid-late to late GF stage (3 and 4 weeks after heading), the greenness index was higher in Takanari than in Koshihikari. There was no significant effect of growth [CO_2_] on greenness index (Table 2).

**Fig. 4:**
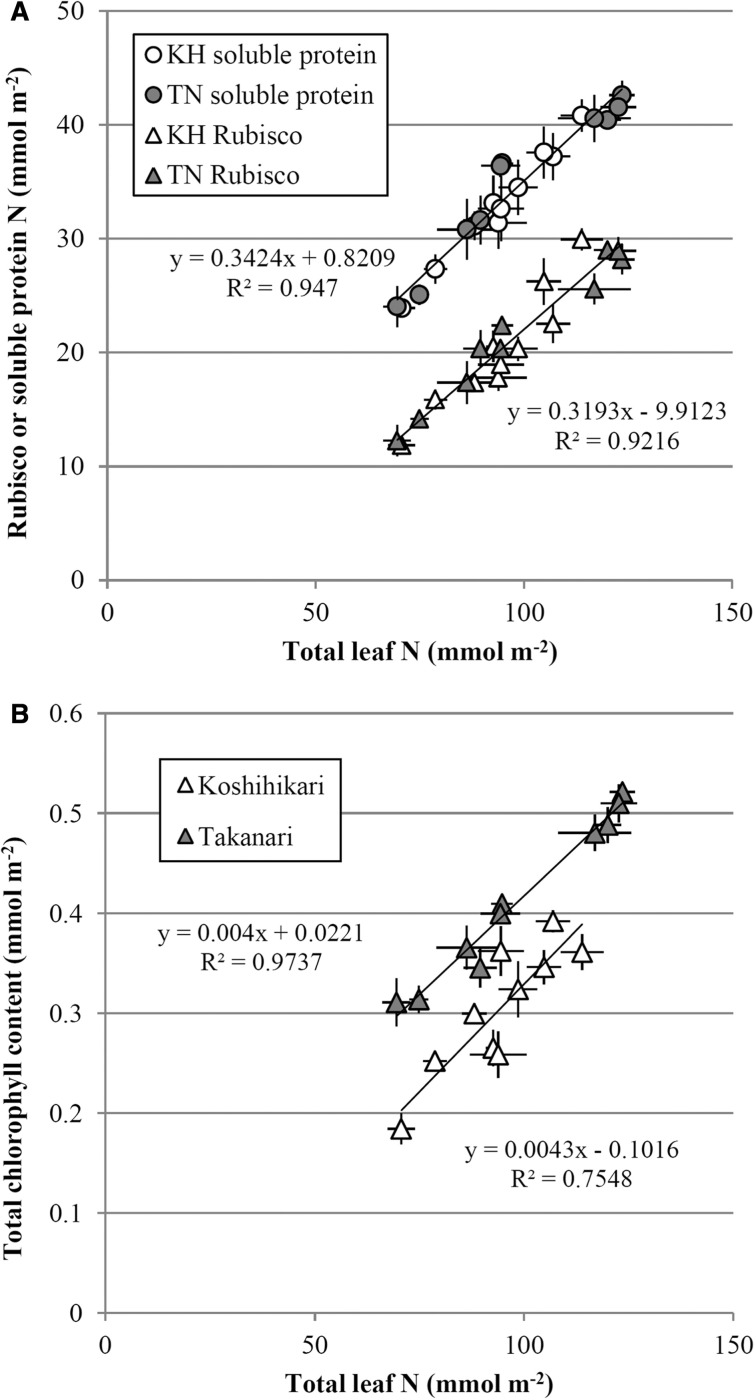
Relationship between total leaf N and Rubisco N, soluble protein N or Chl content, constructed from the time course data in Fig. 3. N content is assumed to be 16% of total protein. (A) Scatterplots of Rubisco N content vs. total leaf N and leaf soluble protein N vs. total leaf N. Circles indicate total soluble protein data, and triangles indicate Rubisco content data. Koshihikari is represented by open symbols, and Takanari is represented by filled symbols. (B) Scatterplot of total Chl content vs. total leaf N. Regression lines in (A) are drawn using pooled data from both cultivars for both ambient- and elevated [CO_2_]-grown treatments (*n* = 20); in (B), separate regression lines are drawn for each cultivar (*n* = 10). Error bars are SEMs of each treatment.

The relationships of Rubisco, leaf soluble protein and Chl to total leaf N were plotted in Fig. 4. All the relationships showed a significant positive linear correlation, and plants grown under both ambient and FACE conditions fell along the same line for each cultivar. The relationships of Rubisco content vs. leaf N and leaf soluble protein vs. leaf N were essentially the same for both cultivars (Fig. 4A). However, Takanari showed a significantly higher Chl content than Koshihikari at the same leaf N level (Fig. 4B; *P* = 0.021).

## Discussion

The results indicate that the source capacity in the flag leaves of Takanari remains higher than that of Koshihikari even when grown in elevated [CO_2_] in the field. Under ambient conditions, the overall leaf carbon assimilation rate, stomatal conductance, biochemical capacity of photosynthesis (*V*_c,max_ and *J*_max_), Chl content and carotenoid content were higher in Takanari compared with Koshihikari on a leaf area basis (Figs. 1–3). The varietal difference in stomatal conductance was particularly striking, as the *g*_s_ of Takanari was at times as high as approximately double that of Koshihikari (Fig. 1B). These results are consistent with earlier reports (Hirasawa et al. 2010, Taylaran et al. 2011), and reflect the high-yield, high-productivity pedigree of Takanari (Takai et al. 2012, Takai et al. 2013). This advantage of Takanari over Koshihikari was maintained when the plants were grown in elevated [CO_2_] under FACE conditions in both 2012 and 2013. Growth in eCO_2_ caused an enhancement in the net carbon assimilation rate of both cultivars to a similar degree (Fig. 1A).

Higher stomatal conductance in Takanari over Koshihikari in ambient conditions resulted in significantly lower overall SL than in Koshihikari in both years (Table 1). The absolute value of SL in the ambient CO_2_ treatment ranged from 6.5% to 13.1% across both years, but the cultivar difference in SL at any given growth stage was only at most 2.2% and 4.0% in the ambient CO_2_ treatment in 2012 and 2013, respectively (Fig. 2D). In the FACE treatment, the absolute value of SL was lower than ambient, ranging from 4.2% to 10.1%, and the maximum cultivar difference of SL in FACE was only 1.6% and 2.3% in 2012 and 2013, respectively.

In contrast, the limitation to photosynthesis due to CO_2_ diffusion from the intercellular airspace to the site of carboxylation (i.e. the MCL) was relatively higher than the SL, ranging from 12.7% to 30.6 % (Fig. 2E). The limitation of photosynthesis by mesophyll conductance was highest at the late GF stage, and it is also here that we observed the biggest cultivar difference in MCL; Takanari had about 36% higher *g*_m_ across both ambient and FACE treatments, resulting in about 7.5% lower MCL than Koshihikari in both ambient and elevated [CO_2_] growth conditions (Fig. 2E). The importance of *g*_m_ in constraining photosynthesis has been explored extensively in the literature (e.g. Niinemets et al. 2009, Adachi et al. 2013), and the ability of Takanari to maintain higher *g*_m_ than Koshihikari late in the season may enable relatively higher rates of leaf photosynthesis even when photosynthetic capacity (*V*_c,max_ and *J*_max_), Rubisco and total leaf N are similar between cultivars (Figs. 2A, B, 3A, C).

In comparison with the ambient [CO_2_] treatment, growth under FACE conditions resulted in lower values of total leaf N, leaf soluble protein content and Rubisco content in Koshihikari as early as the booting stage, and this difference increased as development progressed (Fig. 3A–C). In contrast, Takanari showed no significant difference in leaf N between ambient and elevated [CO_2_] treatments and consequently almost no change in leaf soluble protein content. Since leaf N content is one of the primary determinants of leaf photosynthesis (Evans 1989, Makino 2003), this could be one of the factors driving the ability of Takanari to achieve very high photosynthetic rates while growing under FACE conditions. It is possible that leaf N content does not decrease in Takanari when the plants are grown in eCO_2_ because of its heavy investment in root growth, especially from the panicle initiation stage onwards, and thus possesses a greater capacity for N uptake than Koshihikari during heading (Taylaran et al. 2011). However, this needs to be confirmed through future investigation of the total N uptake and N allocation within the whole plant.

The largest difference between cultivars in Rubisco, soluble protein and total leaf N was observed at the mid GF stage (2 weeks after heading) (Fig. 3A–C), and this coincided with higher *V*_c,max_ and *J*_max_ and higher midday photosynthetic rates in Takanari than in Koshihikari at that growth stage in both 2012 and 2013 (Figs. 1A, 2A, B). This suggests that Takanari may be able to maintain a higher leaf N status and thus higher photosynthetic capacity further into its GF period than Koshihikari, which would contribute to greater overall efficiency of GF before maturation (Nagata et al. 2001). In fact, Takanari shows the ability to fill its inferior spikelets just as well as its superior spikelets, in contrast to Koshihikari (G. Zhang, unpublished data), and our results here indicate a potential mechanism of longer duration of carbon assimilate supply during GF to enable that phenomenon. By the mid-late and late GF stages (3 and 4 weeks after heading), the leaf N, soluble protein and Rubisco content of both ambient-grown and FACE-grown Takanari were similar to those of FACE-grown Koshihikari (Fig. 3A–C). Ambient-grown Koshihikari showed higher values at the last sampling time point, perhaps indicating a slowing down of nutrient translocation into the grain and presence of sink capacity limitation.

Takanari showed a significantly higher pigment content (Chl *a* + *b* and carotenoids) than Koshihikari from as early as the booting stage until near the end of GF (Fig. 3D, E). The Chl content vs. leaf N relationship showed a consistently higher Chl content in Takanari over Koshihikari at the same leaf N content (Fig. 4B), indicating that Takanari invests more of its N in light capture, although the impact of this difference on actual photosynthetic rates is not clear.

**Fig. 5:**
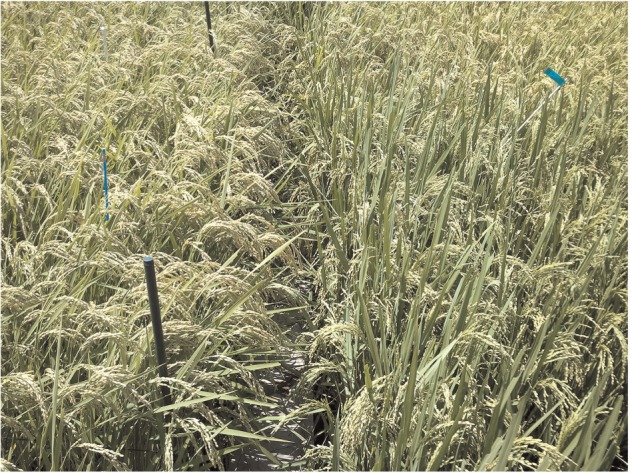
A colour photograph showing the canopies of Koshihikari (left) and Takanari (right) during the mid-grain filling stage.

Why does Takanari keep its flag leaves greener than Koshihikari, even until the late stages of GF (Fig. 3D)? One explanation might be found in the canopy architecture of Takanari, which is distinctly different from that of Koshihikari (Xu et al. 1997b). In Koshihikari, the orientation of the flag leaves is relatively horizontal, and the panicles remain either above or at the same height as the flag leaves after heading, resulting in shading of the flag leaves and lower total interception of radiation as GF progresses (Fig. 5, left side). However, in Takanari, the flag leaves are comparatively wider and taller, and maintain a vertical orientation throughout the GF stage (Fig. 5, right side). This ensures that its flag leaf continues to be exposed to full light even as its large panicles become heavy and sag downwards during GF, and the presence of Chl may enable continued photosynthesis and carbohydrate production even at the later stages of GF, resulting in a high efficiency of GF (Xu et al. 1997a, Nagata et al. 2001, Saitoh et al. 2002).

There is very limited intraspecific variability in the properties of Rubisco in rice, so the potential to increase rice productivity through breeding for direct improvements in the leaf biochemical properties may be small (Makino 2011). Instead, increased stomatal and/or mesophyll conductance, more efficient canopy structure or better optimized within-plant N allocation may be more useful targets for breeding higher productivity in rice (Saitoh et al. 2002, Hirasawa et al. 2010, Makino 2011, Adachi et al. 2013). Our results indicate that Takanari performed well in all of these aspects when grown in elevated [CO_2_] under field conditions, and would be a useful genetic resource for breeding new varieties of higher yielding rice that can thrive in future as well as present atmospheric conditions.

## Materials and Methods

### Site description

We conducted the study at the Tsukuba FACE experimental facility in Tsukubamirai City, Ibaraki Prefecture, Japan (35°58′N, 139°60′E, 10 m a.s.l.) in the summers of 2012 and 2013. The soil is a Fluvisol, which is typical of alluvial areas. The average air temperature at the site in 2012 was 19.8°C in June, 24.9°C in July, 26.8°C in August and 25.5°C for the first half of September; in 2013, it was 21.3, 25.0, 26.9 and 25.1°C, respectively. The average ambient [CO_2_] at the site across the entire growing season (June–September) was 383.1 µmol mol^−1^ in 2012 and 383.7 µmol mol^−1^ in 2013. There were four control (ambient) plots and four FACE plots at the site (*n* = 4). The target concentration of the elevated [CO_2_] treatment (hereafter referred to as FACE treatment) was 200 µmol mol^−1^ above ambient [CO_2_], and the actual season-long mean [CO_2_] in the FACE plots was 577.1 µmol mol^−1^ in 2012 and 576.0 µmol mol^−1^ in 2013. Further details regarding the experimental site set-up and CO_2_ control performance can be found in Nakamura et al. (2012), and soil chemical properties are given in detail in Hasegawa et al. (2013).

Two rice (*Oryza sativa* L.) cultivars were used in this study. ‘Koshihikari’ is a *japonica* variety, and ‘Takanari’ is an *indica* variety. Three-week-old seedlings were transplanted into the experimental plots on May 23–24 in 2012 and May 22–23 in 2013. CO_2_ fumigation was begun in 2012 on May 30 and ran until September 13; in 2013, fumigation ran from May 28 to September 13. In both years, 50% heading for Koshihikari was achieved by August 3, and for Takanari by August 9 and 6 in 2012 and 2013, respectively.

### Gas exchange measurements

Measurements of photosynthesis and stomatal conductance were conducted using a portable photosynthetic gas exchange system (LI-6400, Licor Inc.). Measurements were taken on the uppermost fully expanded leaves (two leaves for each cultivar per experimental plot per time point) at a photosynthetic photon flux density of 1,500–1,800 µmol m^−2^ s^−1^ and leaf chamber temperature of 32°C. The average leaf temperature within the chamber was 34.1 ± 0.9°C and 33.1 ± 0.5°C for Koshihikari and Takanari, respectively. The ambient air in the rice paddy was typically very humid, so the humidity was controlled through the occasional use of desiccant (Drierite, W.A. Hammond Drierite Co. Ltd.), resulting in a relative humidity within the leaf chamber of 79.4 ± 5.6% for Koshihikari and 80.5 ± 2.4% for Takanari. Vapor pressure deficit was 1.36 ± 0.34 kPa for Koshihikari and 1.08 ± 0.14 kPa for Takanari.

CO_2_ response curves, consisting of 12 levels of CO_2_ ranging from 50 to 1,500 µmol mol^−1^, were measured on two leaves per cultivar per ring during the mid GF and late GF growth stages in 2012 (approximately 2 and 4 weeks after each cultivar’s respective heading dates), and during the full heading and mid GF stage in 2013 (3 days and 2 weeks after heading, respectively).

### Estimation of photosynthetic parameters and mesophyll conductance

According to the Farquhar et al. (1980) steady-state biochemical model of leaf photosynthesis, the biochemical capacity for photosynthesis can be defined by two key parameters, namely, *V*_c,max_ and *J*_max_ (Farquhar et al. 1980, von Caemmerer 2013). A third biochemical limitation to photosynthesis may also occur in the form of triose-phosphate utilization limitation (Sharkey 1985), but will not be considered here due to the inability to confirm conclusively its presence in the field study. These parameters can be estimated via curve fitting of the relationship between the net carbon assimilation rate and the mole fraction of CO_2_ within the intercellular airspace of the leaf (*C*_i_) [detailed equations may be found in Long and Bernacchi (2003) and von Caemmerer (2013)]. An Excel Solver-based non-linear curve-fitting utility based on these equations (Sharkey et al. 2007) was used simultaneously to solve for *V*_c,max_, *J*_max_ and *g*_m_ at leaf temperature. This utility employs the curve-fitting method of Ethier and Livingston (2004) to estimate *g*_m_ from gas exchange data. To eliminate temperature differences between individual leaves at the time of measurement, *V*_c,max_ and *J*_max_ were adjusted to 32°C using the temperature response functions of Bernacchi et al. (2001, 2003) which have been implemented in the Sharkey et al. (2007) curve-fitting utility; values of mesophyll conductance were adjusted to 32°C using the temperature response function of Scafaro et al. (2011) for *O. sativa*.

*A–C*_i_ data were also fit under the assumption that *g*_m_ is infinite and *C*_i_ = *C*_c_ to produce estimates of *V*_c,max_ and *J*_max_ from the ‘conventional’ curve-fitting method (described by Long and Bernacchi 2003). We found that this method produced a poorer fit of the measured data points, so only results from the curve-fitting method described above (Sharkey et al. 2007) are used in this study. Example *A–C*_i_ and *A–C*_c_ fitted curves can be found in Supplementary Fig. S1, and the mean parameter estimates derived from the conventional curve-fitting are also available (Supplementary Table S1).

### Calculation of CO_2_ diffusion limitations to photosynthesis

Limitations to photosynthesis due to resistance in the CO_2_ diffusion pathway from the atmosphere to the site of carboxylation were calculated for each *A–C*_i_ curve. This was divided into two components: limitation of photosynthesis due to CO_2_ resistance at the leaf boundary layer and stomata, SL; and limitation of photosynthesis due to resistance from the intercellular airspace to the site of carboxylation, MCL.

SL (expressed as a percentage) was calculated following the method described in Long and Bernacchi (2003):
(1)
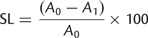

where *A*_0_ represents the theoretical rate of photosynthesis when we assume that the boundary layer conductance to CO_2_, stomatal conductance to CO_2_ and *g*_m_ are infinite, i.e. if *C*_c_ = *C*_a_ (atmospheric mole fraction of CO_2_, assumed to be 390 and 590 µmol mol^−1^ for the ambient and FACE-grown plants, respectively); *A*_1_ represents the theoretical rate of photosynthesis when only *g*_m_ is assumed to be infinite, i.e. if *C*_c_ = *C*_i_.

MCL (expressed as a percentage) was calculated in a similar manner to SL, as follows:
(2)
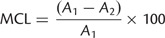

where *A*_1_ is the same as above, and *A*_2_ represents the actual measured photosynthesis rate at *C*_c_ when the measured stomatal conductance to CO_2_ and estimated *g*_m_ are taken into account. A graphical representation of the calculation of SL and MCL in the context of the CO_2_ response curve can be found in Supplementary Fig. S2.

### Biochemical analyses

The uppermost fully expanded leaves of both cultivars were sampled in 2012 at five time points from the booting stage (late July, approximately 1.5 weeks before heading) to the late GF stage (early September, 4 weeks after heading). Three leaves per cultivar were sampled in each experimental plot per time point.

Approximately 6 cm^2^ of leaf material was sampled from the same leaves and immediately frozen in liquid nitrogen and stored at −80°C. After thawing, 0.5 cm^2^ of leaf material was placed in 1.8 ml of 95% ethanol and stored in the dark at 4°C for 3 d to extract the leaf pigments. The absorbance of the solutions at 470, 648.6 and 664.1 nm was then measured in a spectrophotometer (Model U-1800, Hitachi High-Technologies Corp.). The total Chl content, carotenoid content and greenness index (the ratio of total Chl to carotenoid content) were calculated from these absorbance values using the equations of Lichtenthaler and Buschmann (2001). Another 1 cm^2^ of leaf material was ground using a glass mortar and pestle chilled at 4°C with 500 µl of extraction buffer consisting of 50 mM Tris–HCl (pH 7.6), 5% glycerol, 2 mM Na-iodoacetate, 5 mM dithiothreitol and 0.2% (w/v) polyvinylpyrrolidone. Leaf soluble protein was quantified from a portion of this crude extract using a commercial version of the assay of Bradford (1976) (Quick Start Bradford Protein Assay, Bio-Rad Laboratories Inc.).

A 30 µl aliquot of the crude protein extract was mixed with 30 µl of 1% lithium dodecyl sulfate buffer and heated at 97°C for 3 min. Then 6 µl of this mixture was loaded into a 7.5–15% polyacrylamide gradient gel and electrophoresis was conducted at 150 V for 30 min using the XV Pantera MP electrophoresis system (DRC Co., Ltd.). The gel bands were stained using Quick-CBB Plus (Wako Pure Chemical Industries, Ltd.).

Densitometry analysis was performed to estimate the quantity of Rubisco large subunit. Total Rubisco content was calculated as 1.24 times the Rubisco large subunit content, based on the ratio of molecular weights of the large and small subunits. A single stock of bovine standard albumin (A7906, Lot # SLBC0647V, Sigma-Aldrich Co.) was used to create a set of protein standards to approximate the quantity of Rubisco protein in each gel.

Approximately 10 cm^2^ was taken from the middle section of the leaf blade and dried in an oven at 80°C for at least 3 d. The dried leaf tissue was used to quantify total leaf N content using an NC analyzer (Sumigraph NC-22, SCAS Ltd.).

### Statistical analysis

Gas exchange and biochemical data were analyzed for each year as a randomized complete block split-plot design using a generalized linear mixed model and three-way (cultivar × growth [CO_2_] × growth stage) analysis of variance (PROC GLIMMIX, SAS 9.3, SAS Inc.) to examine overall treatment effects, as well as a two-way (cultivar × growth [CO_2_]) analysis of variance at each growth stage to identify treatment mean differences. The FACE treatment was treated as a whole-plot effect, and the cultivars were subplots.

## Supplementary data

Supplementary data are available at PCP online.

## Funding

This work was supported by the Ministry of Agriculture, Forestry and Fisheries, Japan, [through a research project entitled ‘Development of technologies for mitigation and adaptation to climate change in Agriculture, Forestry and Fisheries’]; the Japan Society for the Promotion of Science [a Grant-in-Aid for Scientific Research on Innovative Areas (No.24114711) as part of the project entitled ‘Comprehensive studies of plant responses to high CO_2_ world by an innovative consortium of ecologists and molecular biologists’].

## Supplementary Material

Supplementary Data

## Abbreviations

*A*: net photosynthetic carbon assimilation rate
*C*_a_: atmospheric CO_2_ mole fraction
*C*_c_: CO_2_ mole fraction at the site of carboxylation
*C*_i_: CO_2_ mole fraction in the intercellular airspace
eCO_2_: elevated [CO_2_]
FACE: free-air CO_2_ enrichment
GF: grain filling
*g*_m_: mesophyll conductance to CO_2_
*g*_s_: stomatal conductance to water vapor
*J*_max_: the maximum electron transport rate supporting the regeneration of ribulose-1,5-bisphosphate
MCL: mesophyll conductance limitation to photosynthesis
N: nitrogen
Rubisco: ribulose-1,5-bisphosphate carboxylase oxygenase
SL: stomatal limitation to photosynthesis
*V*_c__,_: _max_, the maximum carboxylation rate of Rubisco
