# Supplementary material for: Do the Rich Always Become Richer? Characterizing the Leaf Physiological Response of the High-Yielding Rice Cultivar Takanari to Free-Air CO2 Enrichment
Source: Plant Cell Physiol. 2014 Jan 30;55(2):381–91. doi: 10.1093/pcp/pcu009 (PMC3913450; doi:10.1093/pcp/pcu009)
Supplement: Supplementary Data [file supp_55_2_381__index.html]

Do the rich always become richer? Characterizing the leaf physiological response of the high-yielding rice cultivar Takanari to free-air CO2 enrichment — Do the Rich Always Become Richer? Characterizing the Leaf Physiological Response of the High-Yielding Rice Cultivar Takanari to Free-Air CO2 Enrichment — Supplementary Data 

# Do the Rich Always Become Richer? Characterizing the Leaf Physiological Response of the High-Yielding Rice Cultivar Takanari to Free-Air CO2 Enrichment

## Supplementary Data

files

**Files in this Data Supplement:**

- Supplementary Data - pdf file
- Supplementary Data - pdf file
- Supplementary Data - pdf file
