# Supplementary material for: Do the Rich Always Become Richer? Characterizing the Leaf Physiological Response of the High-Yielding Rice Cultivar Takanari to Free-Air CO2 Enrichment
Source: Plant Cell Physiol. 2014 Jan 30;55(2):381–91. doi: 10.1093/pcp/pcu009 (PMC3913450; doi:10.1093/pcp/pcu009)
Supplement: Supplementary Data [file supp_pcu009_pcp-2013-e-00573-File007.pdf]

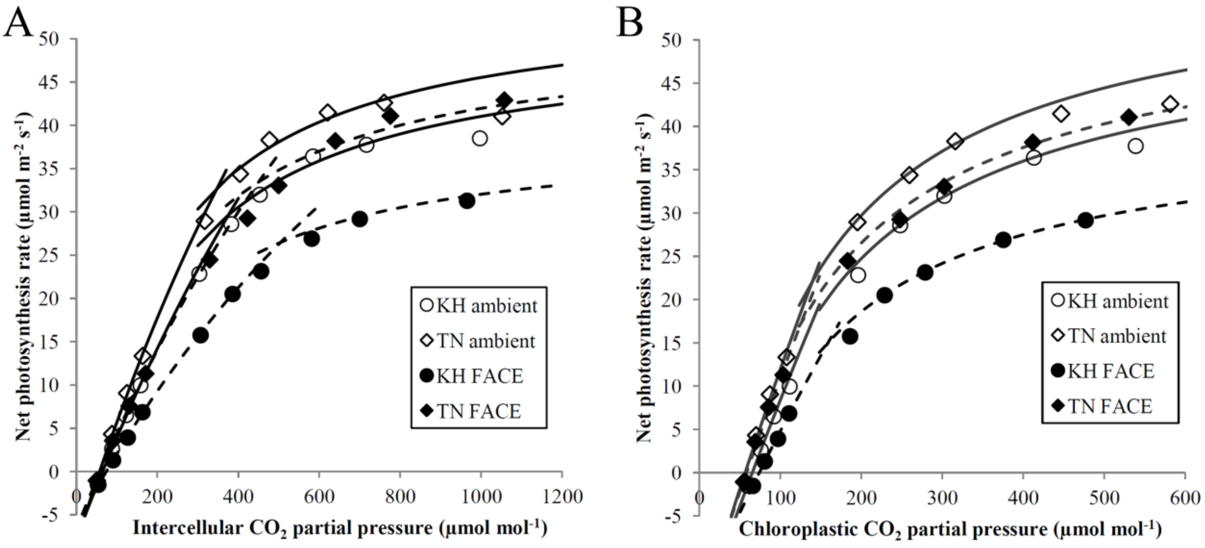

Fig. S1. Example CO_2 response curves of plants measured on flag leaves in the field during the mid-grain filling stage in 2013. Circles and diamonds indicate measurements of Koshihikari and Takanari, respectively. Open and closed symbols indicate plants grown in ambient and FACE conditions, respectively. (a) A-C_i curves fitted using to data points directly measured in the field by a commercial infra-red gas analyzer, using the "conventional method" described by Long and Bernacchi (2003). (b) A-C_c curves calculated from the A-C_i curves in (a) incorporating estimates of mesophyll conductance by employing a curve-fitting utility that simultaneously solves for mesophyll conductance alongside $V_{c,max}$ and J_{max} (Sharkey et al. 2007).
