# Supplementary material for: Do the Rich Always Become Richer? Characterizing the Leaf Physiological Response of the High-Yielding Rice Cultivar Takanari to Free-Air CO2 Enrichment
Source: Plant Cell Physiol. 2014 Jan 30;55(2):381–91. doi: 10.1093/pcp/pcu009 (PMC3913450; doi:10.1093/pcp/pcu009)
Supplement: Supplementary Data [file supp_pcu009_pcp-2013-e-00573-File008.pdf]

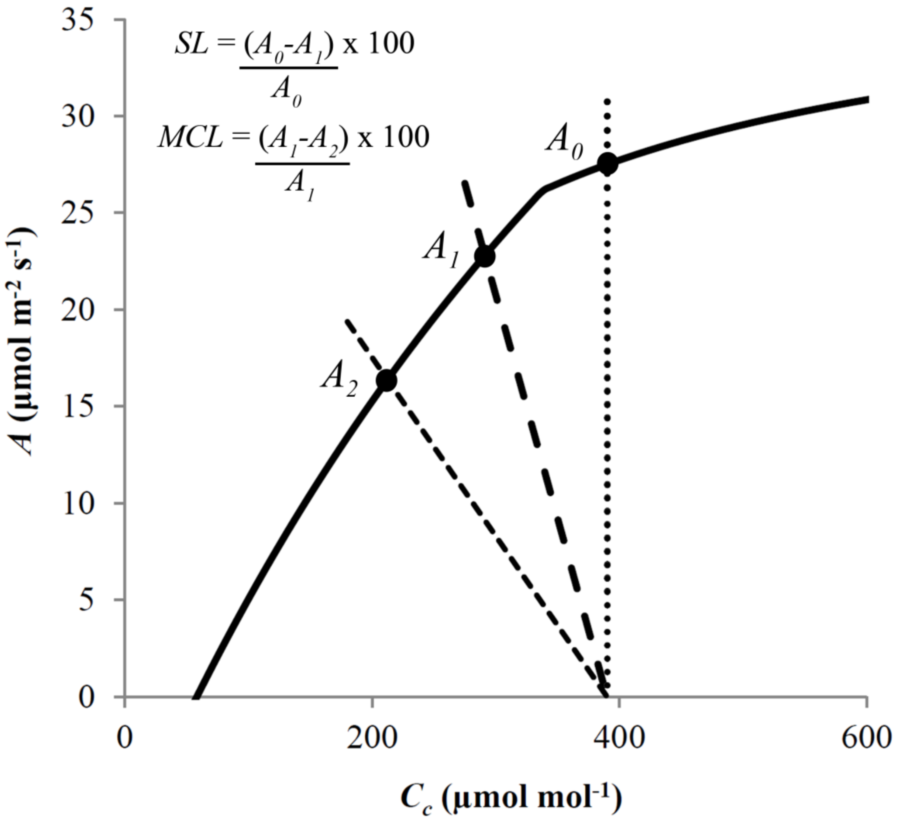

Fig. S2. Graphical representation of the estimation of the limitation to photosynthesis by the stomata (*SL*) and limitation by mesophyll conductance (*MCL*). An idealized CO_2 response curve (A-C_c) is shown as a solid line, where A is the net carbon assimilation rate and C_c is the molar fraction of carbon dioxide at the site of carboxylation. A_0 represents the theoretical rate of photosynthesis when we assume that the boundary layer conductance, stomatal conductance (g_s), and mesophyll conductance (g_m) are infinite, i.e., if $C_c = C_a$ (atmospheric mole fraction of CO_2, in this case 390 µmol mol^{-1}). A_1 represents the theoretical rate of photosynthesis when only g_m is assumed to be infinite, i.e., if $C_c = C_i$ (intercellular mole fraction of CO_2). A_2 represents the actual observed photosynthesis rate when the measured or estimated g_s and g_m are taken into account. The dotted line represents the CO_2 supply curve when there is no resistance to diffusion of CO_2 from the outside of the leaf to the site of carboxylation; the thick-dashed line represents the CO_2 supply curve in the presence of a finite boundary layer and g_s, and the thin-dashed line is the supply curve when g_m is also finite.
