# Supplementary material for: Do the Rich Always Become Richer? Characterizing the Leaf Physiological Response of the High-Yielding Rice Cultivar Takanari to Free-Air CO2 Enrichment
Source: Plant Cell Physiol. 2014 Jan 30;55(2):381–91. doi: 10.1093/pcp/pcu009 (PMC3913450; doi:10.1093/pcp/pcu009)
Supplement: Supplementary Data [file supp_pcu009_pcp-2013-e-00573-File009.pdf]

Table S1. Parameter estimates of $V_{c,max}$ and J_{max} using the "conventional" method of curve-fitting A-C_i data (i.e., assuming g_m is infinite and $C_i = C_c$), as described by Long and Bernacchi (2003). Values are corrected from leaf temperature at measurement to 32°C using the temperature response functions of Bernacchi et al. (2001) and Bernacchi et al. (2003). N=4 for each timepoint.

				**$V_{c,max}$ at 32°C**		**J_{max} at 32°C**	
Year	Stage	CO_2	Cultivar	Mean	SE	Mean	SE
2012	Mid GF	Ambient	Koshihikari	174.70	4.57	262.36	6.79
			Takanari	213.86	13.40	283.76	12.36
		FACE	Koshihikari	161.79	7.09	239.03	14.85
			Takanari	203.92	5.93	283.91	12.32
	Late GF	Ambient	Koshihikari	130.18	6.59	206.77	7.86
			Takanari	149.60	5.94	217.52	9.26
		FACE	Koshihikari	104.60	3.07	177.51	6.59
			Takanari	124.66	5.88	192.24	4.18
2013	Full HD	Ambient	Koshihikari	168.34	10.16	280.90	11.74
			Takanari	206.32	3.66	328.86	12.03
		FACE	Koshihikari	155.93	3.97	244.75	4.85
			Takanari	190.44	5.64	320.75	9.40
	Mid GF	Ambient	Koshihikari	148.87	7.49	230.15	4.32
			Takanari	183.89	6.93	317.42	24.18
		FACE	Koshihikari	123.31	3.94	208.87	4.65
			Takanari	164.83	11.34	286.48	30.14
